# Identification of major hub genes involved in high-fat diet-induced obese visceral adipose tissue based on bioinformatics approach

**DOI:** 10.1080/21623945.2023.2169227

**Published:** 2023-02-01

**Authors:** Yu Jiang, Rui Zhang, Jia-Qi Guo, Ling-Lin Qian, Jing-Jing Ji, Ya Wu, Zhen-Jun Ji, Zi-Wei Yang, Yao Zhang, Xi Chen, Gen-Shan Ma, Yu-Yu Yao

**Affiliations:** a Department of Cardiology, Zhongda Hospital, School of Medicine, Southeast University, Nanjing, P. R. China; b Department of Cardiology, Zhejiang Provincial People’s Hospital, Hangzhou, P. R. China; c Department of Cardiology, Second Affiliated Hospital, College of Medicine, Zhejiang University, Hangzhou, P. R. China; d Department of Cardiology, Anqing First People’s Hospital of Anhui Province, Anqing, P. R. China

**Keywords:** Obesity, high-fat diet, visceral adipose tissue, bioinformatics analysis, inflammation, fibrosis, biomarker

## Abstract

High-fat diet (HFD) can cause obesity, inducing dysregulation of the visceral adipose tissue (VAT). This study aimed to explore potential biological pathways and hub genes involved in obese VAT, and for that, bioinformatic analysis of multiple datasets was performed. The expression profiles (GSE30247, GSE167311 and GSE79434) were downloaded from Gene Expression Omnibus. Overlapping differentially expressed genes (ODEGs) between normal diet and HFD groups in GSE30247 and GSE167311 were selected to run protein–protein interaction network, GO and KEGG analysis. The hub genes in ODEGs were screened by Cytoscape software and further verified in GSE79434 and obese mouse model. A total of 747 ODEGs (599 up-regulated and 148 down-regulated) were screened, and the GO and KEGG analysis showed that the up-regulated ODEGs were significantly enriched in inflammatory response and extracellular matrix receptor interaction pathways. On the other hand, the down-regulated ODEGs were involved in metabolic pathways; however, there were no significant KEGG pathways. Furthermore, six hub genes, Mki67, Rac2, Itgb2, Emr1, Tyrobp and Csf1r were acquired. These pathways and genes were verified in GSE79434 and VAT of obese mice. This study revealed that HFD induced VAT expansion, inflammation and fibrosis, and the hub genes could be used as therapeutic biomarkers in obesity.

## 1. Introduction

Obesity is a worldwide threatening public health problem and is a crucial risk factor in several metabolic-related diseases [1,2]. High-fat and high-calorie diets are common unhealthy lifestyles that contribute to a significant impact worldwide and affect all ages[3].

Obesity is characterized by weight gain accompanied by excessive fat accumulation, and the expansion of white adipose tissue (WAT) is a mutual result of adipocyte hyperplasia and hypertrophy[4]. It has been described that WAT is composed of adipocytes and several non-adipocyte populations, designed as a stromal vascular fraction (SVF), including adipocyte precursors, vascular component cells, immune cells, fibroblasts and extracellular matrix (ECM) components[5]. Furthermore, cellular interactions in adipocytes and SVF can contribute to a complex WAT microenvironment. Some reports demonstrated that a stable WAT microenvironment is essential to maintain metabolic homoeostasis, and high-fat diet (HFD) can induce a microenvironmental disturbance in an early stage, in visceral adipose tissue (VAT) [6,7]. Moreover, half a century ago, Jean Vague revealed that obese individuals who prioritized VAT expansion had a higher risk of developing metabolic disorders than obese individuals with subcutaneous adipose tissue accumulation[8]. An obese mouse model fed with a HFD could be widely used to study the pathophysiological process of obesity-related metabolic diseases. However, the gene networks involved in obese VAT are complex and remain unclear[9]. Therefore, clarifying the major pathways and genes involved in obese VAT caused by HFD could be relevant to understand the biological mechanisms associated with obesity. Furthermore, this study can be valuable to help identify therapeutic biomarkers in obesity.

**Figure 1.**
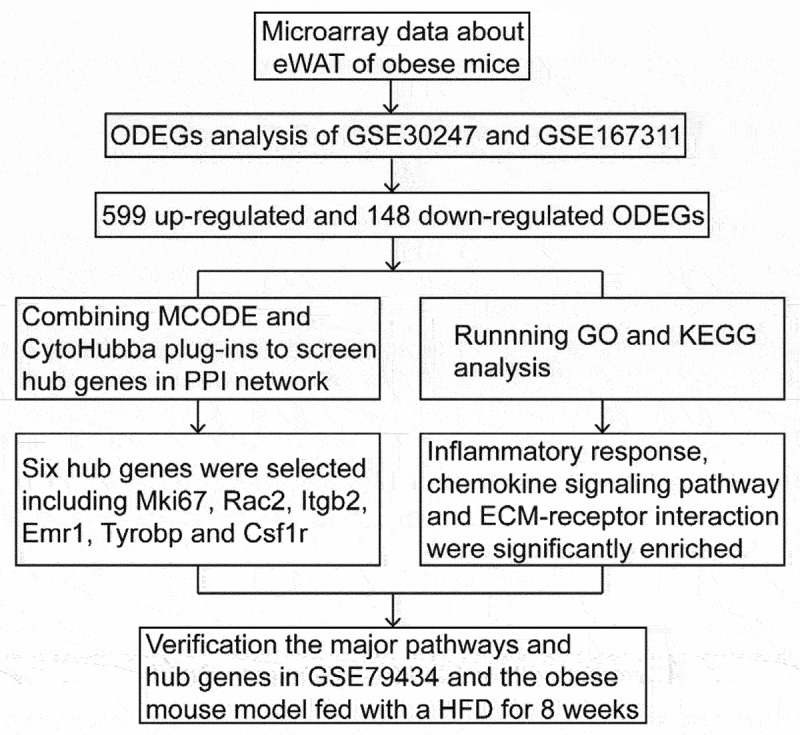
A schematic view of the study’s procedure that combining with the analysis between GSE30247 and GSE167311, selecting major pathways and hub genes to validate in GSE79434 and the obese mouse model. eWAT: epididymal white adipose tissue; ODEGs: overlapping differentially expressed genes; MCODE: Molecular Complex Detection; PPI: protein–protein interaction; GO: Gene Ontology; KEGG: Kyoto Encyclopedia of Genes and Genomes; Mki67: monoclonal antibody Ki 67; Rac2: Rac family small GTPase 2; Itgb2: integrin beta 2; Emr1: emerin homolog 1; Tyrobp: TYRO protein tyrosine kinase binding protein; Csf1r: colony-stimulating factor-1 receptor; EMC: extracellular matrix; HFD: high fat diet.

Recently, gene expression profile analysis combined with bioinformatics has become a general and effective methodology to investigate vital signalling pathways and genes involved in various diseases. Therefore, analysing bioinformatics databases can help to predict, diagnose and treat several diseases [10,11]. Although single-cell sequencing has unique advantages and has become an ideal tool for single-cell research because of its high accuracy and specificity, and it can sequence genome, transcriptome and epigenome at the single-cell level. However, this is precisely the deficiency of single-cell sequencing, as it cannot reflect the comprehensive effects of multiple cells in tissue. Therefore, microarray data is a necessary and important analysis source, which can guide the research at the tissue level. This study was performed using a multi-step strategy (Figure 1). Three independent microarray datasets of epididymal white adipose tissue (eWAT) in obese mice were enrolled for research. Then, the overlapping differentially expressed genes (ODEGs) between normal diet (ND) and HFD groups of two datasets were screened. Gene Ontology (GO) and Kyoto Encyclopedia of Genes and Genomes (KEGG) were applied to unveil the role of ODEGs. Additionally, protein–protein interaction (PPI) network was employed to select the hub genes of ODEGs. Moreover, the major pathways and hub genes found in this study were validated using a third dataset and eWAT of obese mice.

## 2. Results

### 2.1. GSE data quality assessment

**Figure 2.**
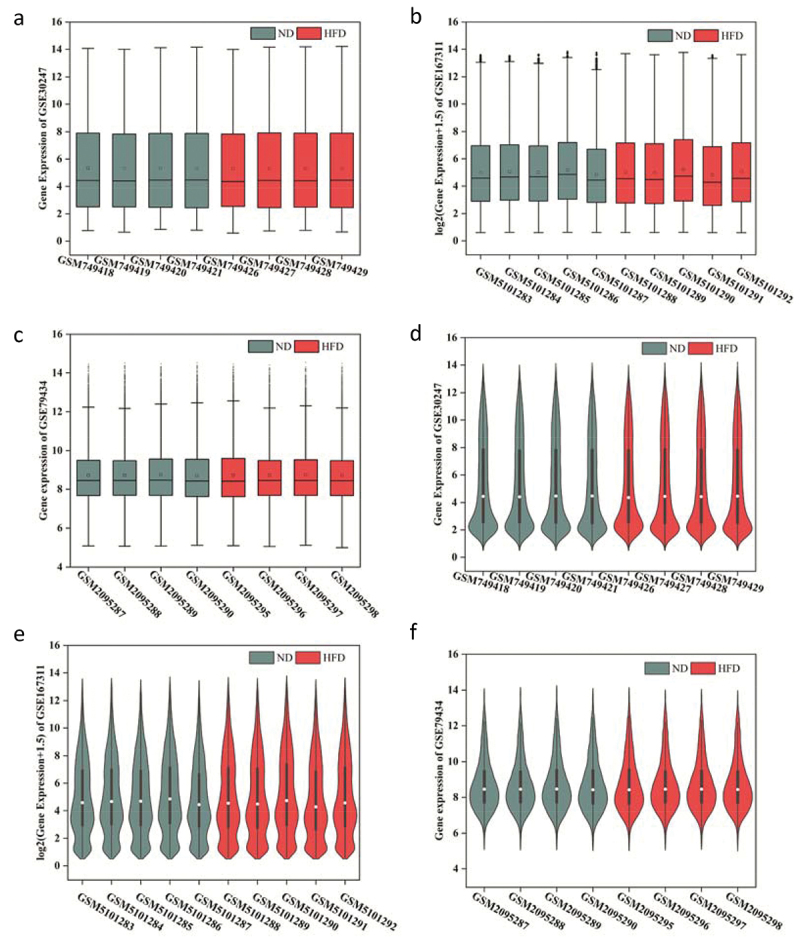
Quality assessment of the GSE data. (a-c) Boxplot charts for the standardized data of GSE30247, GSE167311 and GSE79434; (d-f) Vioplot charts for the standardized data of GSE30247, GSE167311 and GSE79434. The X axis contains the name of each sample, and the Y axis showed the gene expression levels for each sample. ND: normal diet.

After downloading the raw materials of GSE30247, GSE167311 and GSE79434 from GEO database, the data quality was assessed, and the boxplot and vioplot were constructed for data standardization (Supplementary File 1–3). The results from the boxplot demonstrated that the samples containing median, upper, and lower quartiles were similar in GSE30247, GSE167311 and GSE79434 (Figure 2a,c). Additionally, it was possible to observe that the density trends of most gene expression levels were consistent in GSE4648, GSE60993 and GSE79434 (Figure 2d,f).

### 2.2. Screening DEGs

**Figure 3.**
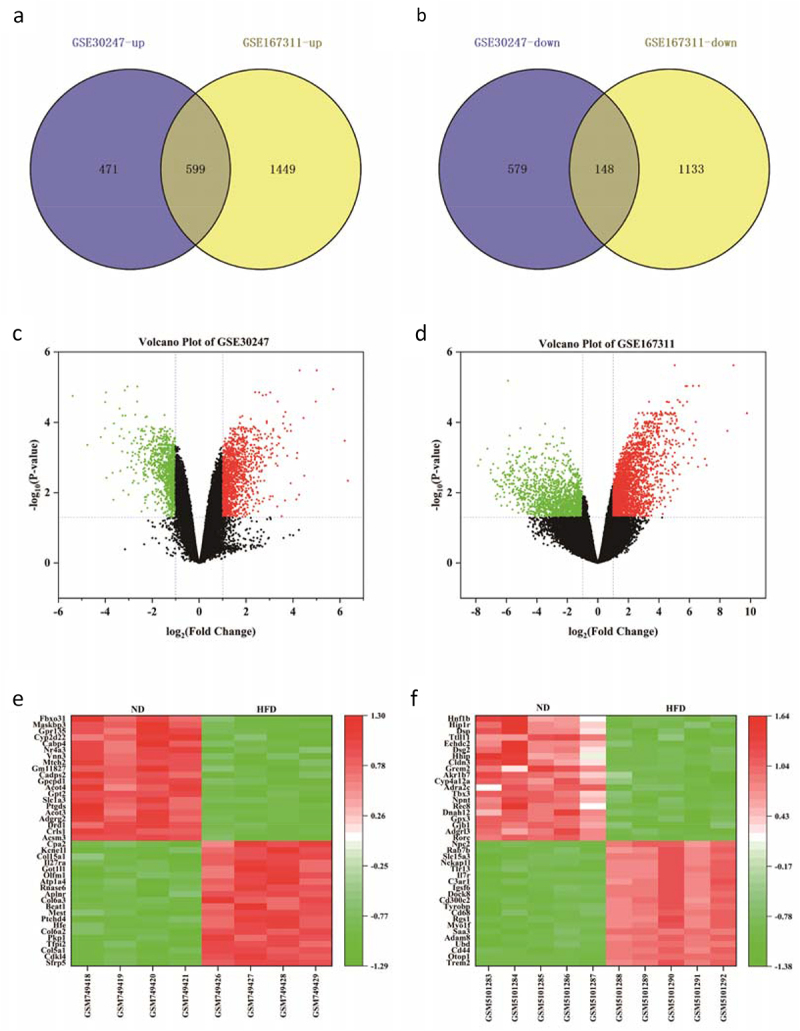
Volcanic and heat maps of DEGs in GSE30247 and GSE167311. (a, b) Venn diagram describing the up and down-regulated ODEGs; (c, d) Volcanic maps of all the respective genes in GSE30247 and GSE167311. Red spot: up-regulated; Green spot: down-regulated; Black spot: no significant difference; (e, f) Respective heat maps for top 40 altered genes of DGEs in GSE30247 and GSE167311. DEGs: differentially expressed genes.

In this study, some parameters were defined to screen DEGs, including |log2 (fold change) | > 1 and FDR < 0.05. According to these criteria, the up-regulated and down-regulated genes between HFD and ND groups in GSE30247 and GSE167311 were evaluated, and a Venn diagram was performed to measure the intersection in these ODEGs. In this study, 747 ODEGs composed of 599 up-regulated and 148 down-regulated ODEGs were identified in GSE30247 and GSE167311 (Figure 3a,b). In view of the fact that the feeding environment was not completely consistent, the individual differences of organisms and the limitation of sample size, the sequencing results conducted by different researchers were varied, so the number of ODEGs obtained was usually limited (Supplementary File 4). Additionally, a volcanic map was constructed to identify the DEGs in GSE30247 and GSE167311. After analysis, more up-regulated genes were detected compared to down-regulated genes in the HFD group (Figure 3c,d). Furthermore, a heatmap was constructed with the top 40 significantly altered genes in GSE30247 and GSE167311 to detect DEGs (Figure 3e,f’).

### 2.3. GO and KEGG functional analysis

**Figure 4.**
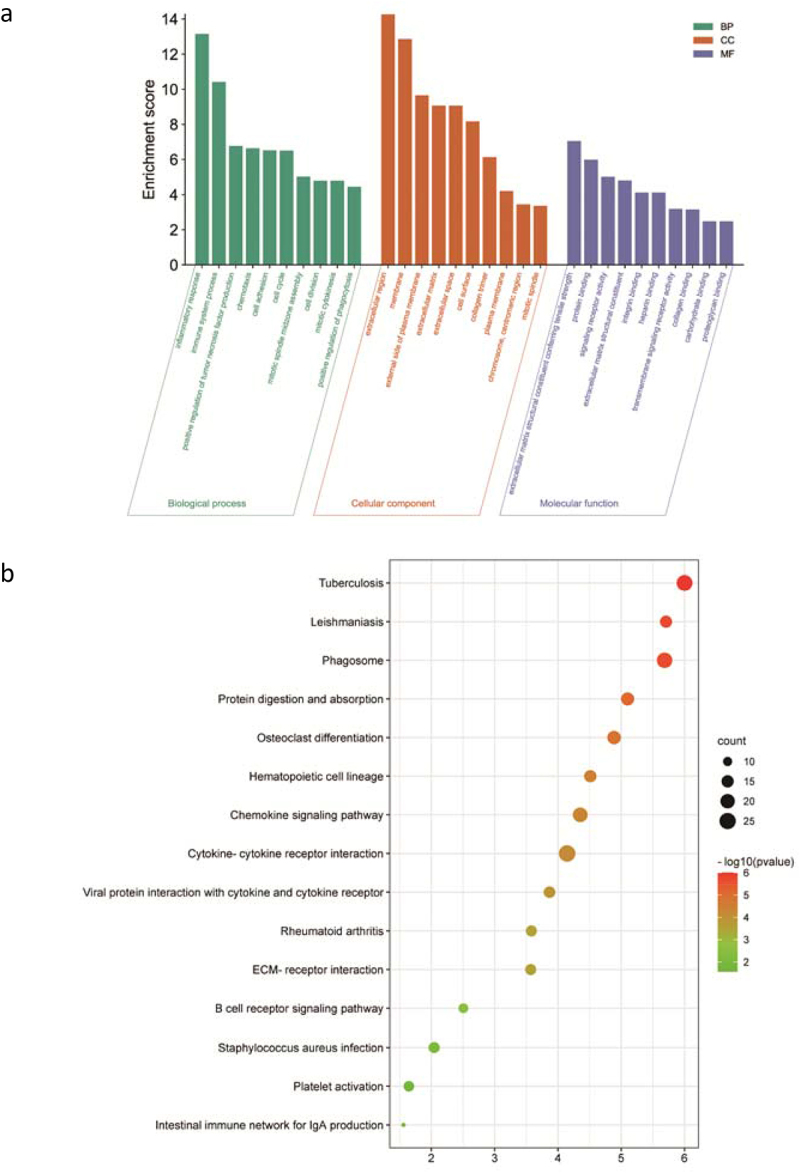
GO and KEGG analysis for the up-regulated ODEGs. (a) GO analysis for the up-regulated ODEGs: biological process (BP), cellular component (CC) and molecular function (MF); (b) KEGG analysis for the up-regulated ODEGs. FDR < 0.05 was considered significant.

**Figure 5.**
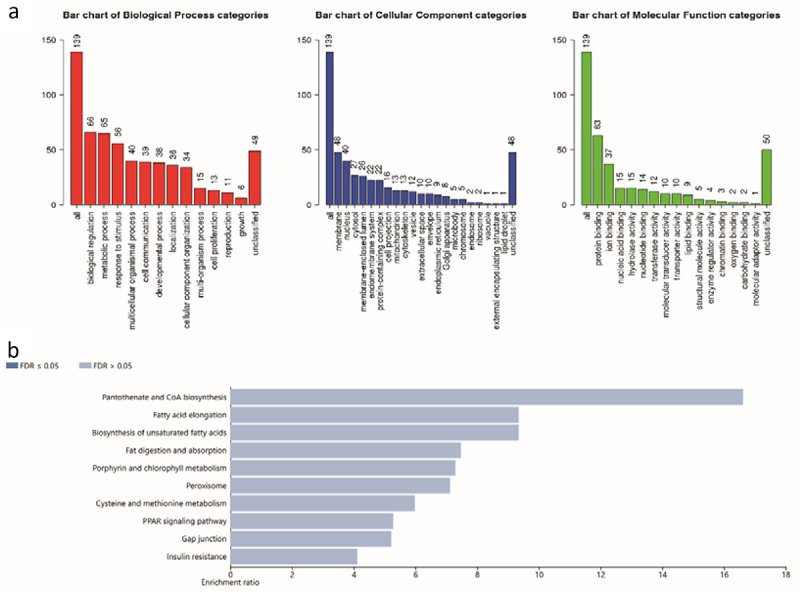
GO and KEGG analysis for the down-regulated ODEGs. (a) GO analysis for the down-regulated ODEGs; (b) KEGG analysis for the down-regulated ODEGs. FDR < 0.05 was considered significant.

To explore the biological functions of the ODEGs, GO and KEGG analysis was performed using the online websites DAVID and WebGestalt. The results showed that, for GO terms, the up-regulated ODEGs were involved in different biological pathways, including inflammatory response, immune system process, positive regulation of tumour necrosis factor production and chemotaxis. Additionally, the up-regulated genes were localized mainly in the extracellular region and cellular membrane. Furthermore, these up-regulated genes were molecularly involved in the structural construction of the extracellular matrix, conferring tensile strength and protein binding. On the other hand, the results obtained from KEGG analysis showed that the up-regulated ODEGs were significantly enriched in some obesity-related pathways, such as the chemokine signalling pathway, cytokine–cytokine receptor interaction, and ECM–receptor interaction (Figure 4a,b). Down-regulated ODEGs were also used for GO and KEGG analysis, and the results demonstrated that these genes were mainly involved in metabolic-related pathways. However, there were no significant KEGG pathways enriched in the down-regulated ODEGs (Figure 5a,b).

### 2.4. PPI network of ODEGs and hub genes

**Figure 6.**
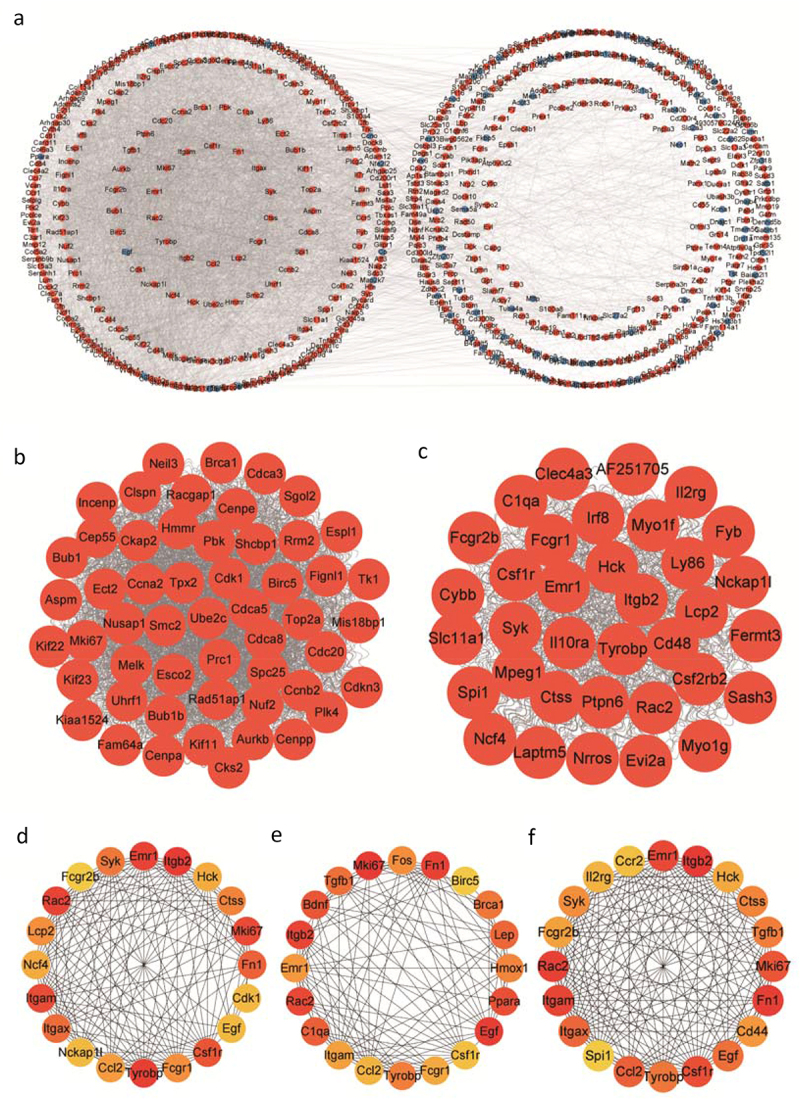
Using the MCODE and cytoHubba plug-ins to search for hub genes in the PPI network of ODEGs. (a) PPI network of ODEGs containing 602 nodes and 5352 edges. Red circle: up-regulated; Blue circle: down-regulated; (b, c) The top 2 modules with the highest score; (d-f) The top 20 genes for each of the three calculation methods (closeness, degree and betweenness).

**Figure 7.**
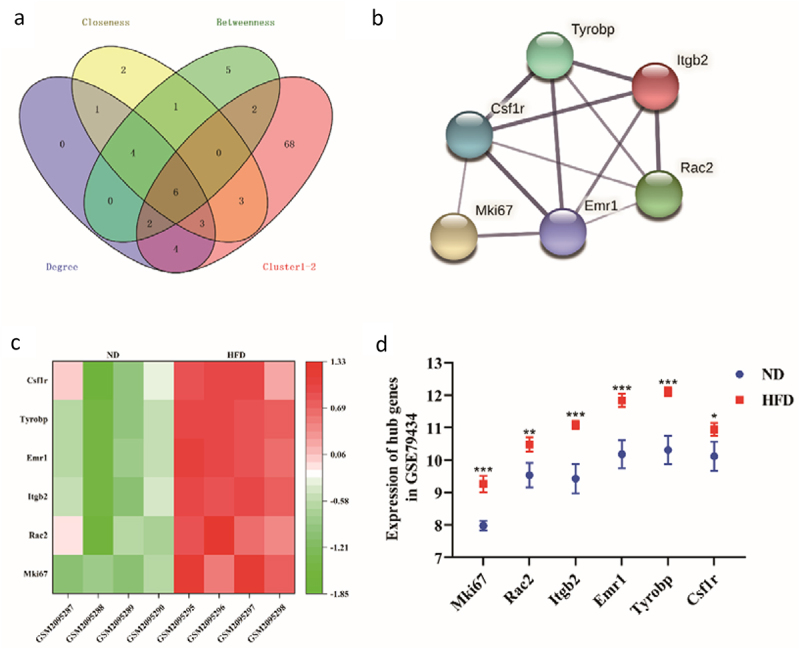
Verification in GSE79434. (a) Venn diagram describing the genes from the top 2 modules module with the highest score intersecting with the top 20 genes from each of the three calculation methods; (b) The PPI network of hub genes; (c) Heatmap of hub genes expression in GSE79434; (d) Boxplot of hub genes expression in GSE79434. (*p < 0.05, **p < 0.01, ***p < 0.001 vs ND group).

In this study, a PPI network composed of 747 ODEGs (602 nodes and 5352 edges) was constructed to understand the interactions of the ODEGs. Then, MCODE plug-in was performed to obtain cluster function modules in the complex PPI networks. The results revealed that the top 2 modules with the highest score were 51.654 (53 nodes and 1343 edges) and 28.412 (35 nodes and 483 edges) (Figure 6a,c). Additionally, to identify the highly connected genes in this PPI network, cytoHubba plug-in was performed, and the top 20 genes were selected in each calculation method (closeness, degree and betweenness) (Figure 6d,f). The genes from the top 2 modules module with the highest score were intersected with the top 20 genes for each of the three calculation methods, and six hub genes were identified: monoclonal antibody Ki 67 (Mki67), Rac family small GTPase 2 (Rac2), integrin beta 2 (Itgb2), emerin homolog 1 (Emr1), TYRO protein tyrosine kinase binding protein (Tyrobp) and colony-stimulating factor-1 receptor (Csf1r). Finally, a PPI network of hub genes was constructed to reflect their interaction (Figure 7a,b).

### 2.5. Hub genes and major pathways verification in GSE79434

The results showed that the expression levels of the six hub genes in GSE79434 were higher in the HFD group than in the ND group (Figure 7c,d). Furthermore, the GO and KEGG analysis of the up-regulated DEGs in GSE79434 showed that these genes were mainly enriched in inflammatory and chemotaxis-related pathways, which were in agreement with the pathways found in GSE30247 and GSE167311 (Table 1; Table 2).

**Table 1.** GO analysis of DEGs in GSE79434.

Category	Pathways
Biological Process	GO:0006954~ inflammatory response
	GO:0002376~ immune system process GO:0032760~ positive regulation of tumour necrosis factor production GO:0030593~ neutrophil chemotaxis GO:0031663~ lipopolysaccharide-mediated signalling pathway
	GO:0006935~ chemotaxis GO:0032755~ positive regulation of interleukin-6 production GO:0050766~ positive regulation of phagocytosis GO:0032720~ negative regulation of tumour necrosis factor production GO:0070374~ positive regulation of ERK1 and ERK2 cascade GO:0042590~ antigen processing and presentation of exogenous peptide antigen via MHC class I
Cellular Component	GO:0016020~ membrane GO:0009897~ external side of plasma membrane GO:0009986~ cell surface GO:0005886~ plasma membrane GO:0016021~ integral component of membrane GO:0045335~ phagocytic vesicle GO:0005925~ focal adhesion GO:0005764~ lysosome GO:0015629~ actin cytoskeleton GO:0005576~ extracellular region GO:0005768~ endosome
Molecular Function	GO:0004888~ transmembrane signalling receptor activity GO:0031726~ CCR1 chemokine receptor binding GO:0005178~ integrin binding GO:0038023~ signalling receptor activity GO:0048020~ CCR chemokine receptor binding GO:0005515~ protein binding GO:0042056~ chemoattractant activity GO:0008009~ chemokine activity GO:0050839~ cell adhesion molecule binding GO:1990782~ protein tyrosine kinase binding GO:0005114~ type II transforming growth factor beta receptor binding

**Table 2.** KEGG analysis of DEGs in GSE79434.

Category	Pathways
KEGG	mmu04380:Osteoclast differentiation
	mmu04145:Phagosome
	mmu04142:Lysosome
	mmu05323:Rheumatoid arthritis
	mmu05152:Tuberculosis
	mmu04062:Chemokine signalling pathway
	mmu04640:Haematopoietic cell lineage
	mmu04061:Viral protein interaction with cytokine and cytokine receptor
	mmu04620:Toll-like receptor signalling pathway
	mmu05140:Leishmaniasis
	mmu04060:Cytokine-cytokine receptor interaction

### 2.6. Hub genes verification in obese mouse model

**Figure 8.**
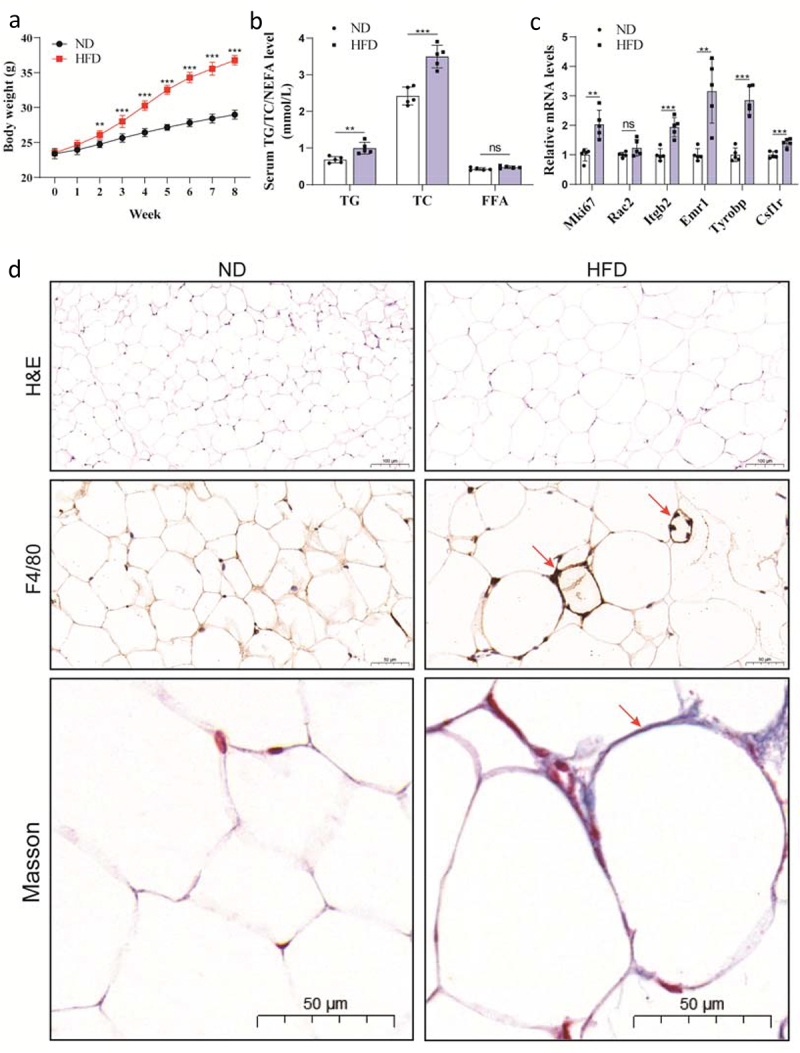
Establishment of the obese mouse model fed with the HFD for 8 weeks. (a) Weekly body weight; (b) Serum TG, TC and NEFA; (c) Validation of six hub genes in obese eWAT using qRT-PCR; (d) Sections of eWAT with H&E staining, immunohistochemistry F4/80 staining (Red arrow: CSLs) and Masson’s trichrome staining (Blue-purple: collagenous connective tissue fibres). TG: triglyceride; TC: total cholesterol; NEFA: non-esterified fatty acid; CSLs: crown-like structures. All data are expressed as mean ± SD. (*p < 0.05, **p < 0.01, ***p < 0.001 vs ND group, n = 5).

The body weight of mice increased significantly after 2 weeks of being submitted to the HFD. Furthermore, the results showed that the average weight of mice fed with a HFD for 8 weeks reached 36.78 ± 0.68 g. On the other hand, mice fed with a ND reached 28.98 ± 0.64 g. Therefore, the body weight in the HFD group was 26.92% higher than in the ND group (Figure 8a), suggesting that the obese mouse model was established and could be used to verify the data. The HFD group exhibited significantly higher serum TG and TC levels than the ND group. However, no significant differences were detected in the NEFA levels between these two groups (Figure 8b). In addition, the validation results of the six hub genes showed that the mRNA levels of Mki67, Itgb2, Emr1, Tyrobp and Csf1r in the HFD group were significantly increased than in the ND group. Rac2 mRNA had an upward trend, but no significant differences were observed between the two mice groups, indicating that a higher number of samples should be used in further studies to confirm this result (Figure 8c).

Besides, in the HFD group, H&E staining showed an increase in the adipocyte size. Masson’s trichrome staining revealed an increase in the collagen, and the immunohistochemical staining of F4/80 showed a higher macrophage infiltration with the formation of a unique histological structure designed as crown-like structures (CSLs) (Figure 8d). These results suggest that eWAT expansion in obesity is accompanied by fibrosis and inflammation development.

## 3. Discussion

This study analysed the ODEGs between two microarray datasets of obese eWAT (GSE30247 and GSE167311). The analysis revealed 747 ODEGs composed of 599 up-regulated and 148 down-regulated ODEGs. GO and KEGG analysis showed that the up-regulated ODEGs were significantly enriched in inflammatory response and fibrosis pathways. On the other hand, the down-regulated ODEGs were mainly involved in metabolic-related pathways. However, there were no significant KEGG pathways. Furthermore, six hub genes, Mki67, Rac2, Itgb2, Emr1, Tyrobp and Csf1r, were obtained after the PPI network analysis. In this study, the enriched pathways in ODEGs were also confirmed using another dataset, GSE79434, and obese mouse model. The results demonstrated that obese eWAT had an increase in adipocyte size, inflammation and fibrosis.

Although these hub genes were independently found to be upregulated in obese VAT in different experiments, it was still unknown whether they were all up-regulated at the early stage of obesity. This study proved that these six hub genes were collectively upregulated at the early stage of obesity (8 weeks of HFD). Mki67, one of the hub genes found in this study, is a marker of cell proliferation, and it has been found to have higher expression levels in SVF during obesity in response to adipose tissue expansion[12]. Furthermore, the other identified five hub genes were involved in inflammation response pathways. For example, Rac2 belongs to a subfamily of Ras homology (Rho) small GTPases. A previous study demonstrated that Rac2 activation is essential in adjusting chemotaxis and reactive oxygen species production in phagocytic cells, including neutrophils and macrophages, by regulating NADPH oxidase[13]. Therefore, Rac inhibition has been associated with better outcomes in metabolic syndrome in obese mice[14]. Additionally, it has been described that Itgb2 is expressed in leukocytes, modulating the adhesion of leukocytes to endothelial cells and promoting the recruitment of leukocytes to inflammatory regions[15]. A previous clinical trial revealed that the Itgb2 expression levels in SVF of obese individuals were significantly higher than in non-obese individuals[16]. Emr1 also known as F4/80 is a marker of macrophage. Elevated Emr1 in obese adipose tissue indicated increased macrophage infiltration[17]. Tyrobp is a transmembrane adaptor protein expressed in several immune cells, including T cells, B cells and macrophages, and plays an essential role activating these cells[18]. It has been described that Tyrobp was up-regulated in obese adipose tissue and decreased its expression levels after bariatric surgery[19]. Additionally, Csf1r is a class III receptor tyrosine kinase that belongs to the platelet-derived growth factor receptor family. CSF1R can be found in mononuclear phagocytic cells and can modulate the development, differentiation and activation of these types of cells[20]. A previous report demonstrated that Csf1r activation was positively correlated with macrophage infiltration and Csf1r inhibition can reduce adipocyte hypertrophy in HFD mice[21]. Overall, these six hub genes were associated with WAT expansion and immune cell infiltration.

WAT remodelling occurs during obesity and is usually characterized by adipocyte hypertrophy, immune cell infiltration, fibrosis and angiogenesis[22]. Chronic low-grade inflammation of WAT caused by obesity is the origin of chronic inflammation in metabolic syndrome. It has been described that substantial immune cells are involved in WAT inflammation, and macrophages are the most abundant and characteristic immune cells in this metabolic disorder[23]. Several reports have revealed that the chemokine ligand and its receptors are essential for the recruitment of macrophages during WAT, mainly the chemokine C–C motif ligand 2 (CCL2)/C–C chemokine receptor 2 (CCR2) axis [24,25]. Adipocyte death due to metabolic stress is a universal biological phenomenon usually during obesity development. Furthermore, previous studies demonstrated that toxic lipids released by dead adipocytes could activate macrophages. These macrophages can phagocytize dead or dying adipocytes, forming CSLs that continuously release inflammatory cytokines and consequently induce WAT inflammation and adipocyte death [26,27]. Therefore, the immune response and inflammation are constantly induced in adipocytes and macrophages.

WAT fibrosis is characterized by excessive accumulation of EMC. The ECM is a non-cellular component mainly composed of proteoglycans and the fibrous proteins, the most abundant of which is collagen. Most collagen is expressed by fibroblasts, myofibroblasts, adipocyte progenitors and adipocytes. Other cell types, such as macrophages, also contribute to ECM production[28]. It has been described that under physiological conditions, the ECM could maintain the structural integrity of the adipocytes and modulate cell–cell communication. However, in obesity-induced WAT fibrosis, there is the accumulation of ECM, leading to the formation of collagen bundles that stiffens WAT[29]. Moreover, WAT fibrosis can limit the excessive expansion of adipocytes, exerting mechanical stress on adipocytes, inhibiting the fat storage capacity of WAT, and finally leading to ectopic lipid deposition[30]. WAT inflammation and fibrosis are dependent processes, and they can interact through several biological mechanisms, eventually leading to WAT dysfunction and systemic metabolic disorders[29]. However, the sequence and causality of these biological events remain unclear.

## 4. Methods

### 4.1. Data origin and collection

GSE is a dataset, which can be obtained by entering keywords in a public high-throughput gene expression database: Gene Expression Omnibus (GEO, https://www.ncbi.nlm.nih.gov/geo). A GSE can contain one or more GSM samples. Each GSE represents the data of an independent sample and each sample has a GSE ID. In this study, each GSE dataset contained GSE samples of ND and GSE samples of HFD. Using HFD and adipose tissue as search keywords, we screened three datasets about HFD for 8 weeks feeding with 60% calories from fat. The basic information of the datasets were shown in Supplementary Table S1. Microarray data of GSE30247 (https://www.ncbi.nlm.nih.gov/geo/query/acc.cgi?acc=GSE30247), GSE167311 (https://www.ncbi.nlm.nih.gov/geo/query/acc.cgi?acc=GSE167311) and GSE79434 (https://www.ncbi.nlm.nih.gov/geo/query/acc.cgi?acc=GSE79434) were acquired from GEO database a public high-throughput gene expression database: Gene Expression Omnibus (GEO, https://www.ncbi.nlm.nih.gov/geo). These databases were applied to analyse the gene expression matrixes in eWAT of C57BL/6 mice fed the ND or HFD for 8 weeks.

### 4.2. ODEGs analysis

Firstly, using the Origin 2021 software, a boxplot and vioplot analysis was performed to evaluate the quality of each microarray data. Then, differentially expressed genes (DEGs) between the ND and HFD groups in GSE30247 and GSE167311 were selected using the GEO2R online data analysis tool (https://www.ncbi.nlm.nih.gov/geo/geo2r/) which was based on GeoQuery and Limma R packages. DEGs were obtained using the formula |log2 (fold change) | > 1. The false discovery rate (FDR) was adjusted, and a *p*-value < 0.05 was used as cut-off criteria. After deleting probes matching multiple genes, the DGEs were plotted in volcanic maps, and the top 40 significant DGEs were used to construct the corresponding heatmap graphics. Additionally, a Venn diagram defined the intersection of up-regulated or down-regulated ODGEs between GSE30247 and GSE167311(https://bioinfogp.cnb.csic.es/tools/venny/index.html).

### 4.3. GO and KEGG analysis

GO and KEGG analyses were performed for functional gene annotation. The GO terms are mainly composed of three biological categories: biological process (BP), cellular component (CC) and molecular function (MF). In this study, an online website tool designed as WebGestalt (http://www.webgestalt.org/) was applied to analyse up-regulated and down-regulated ODEGs. Finally, significant enrichment was determined when FDR was lower than 0.05.

### 4.4. PPI network

An online website designed as STRING (https://string-db.org/) was used to establish a PPI network to analyse the internal connection between the ODEGs. Some parameters were defined to construct this PPI network: the minimum interaction score was greater than 0.4, and the unconnected nodes were removed from the analysis. Subsequently, the obtained PPI network was imported to the Cytoscape 3.9.1 software. The hub genes were selected by two Cytoscape plugins: Molecular Complex Detection (MCODE) and CytoHubba. The top 20 genes from three approaches using the cytoHubba plug-in were extracted: betweenness, degree and closeness. In this study, the hub genes were obtained by intersecting the genes from three approaches with genes in the top 2 modules module with the highest MCODE score. Finally, a Venn diagram was constructed to analyse the data.

### 4.5. Verification of major pathways and hub genes in GSE79434

As previously described, up-regulated DEGs between the ND and HFD groups in GSE79434 were screened and performed GO and KEGG analysis. Furthermore, the expression levels of hub genes in GSE79434 were investigated using heatmap and boxplot drawing.

### 4.6. Animal research

#### 4.6.1 Animal

The animal procedures were performed accordingly to the China National Standards and Guidelines for Laboratory Animal Management and were approved by the Animal Care and Use Committee of Southeast University (No.: 20200326003). In this study, ten 7-week-old male C57BL/6 mice were purchased from the Changzhou Cavens Laboratory Animal Ltd. (Changzhou, China). These animals were housed at room temperature and exposed to a 12-hour light/dark cycle and free access to food and water. After a week of ND feeding adaptation, half of these mice (n = 5/group) were submitted to a HFD containing 60% kcal of fat from Xietong Pharmaceutical Bio-engineering Co., Ltd. (Jiangsu, China) for 8 weeks. Additionally, the body weight of the two groups was measured regularly every week.

#### 4.6.2 Serum lipids determination

After 8 weeks of exposure to these diets, the mice experienced fasting overnight and were anesthetized by intraperitoneal pentobarbital (50 mg/kg) injection, and whole blood was collected via the retro-orbital sinus plexus. This blood was then centrifuged to obtain serum. Finally, after obtaining the serum from these mice, the total cholesterol (TC), triglyceride (TG) and non-esterified fatty acid (NEFA) levels were determined by enzymatic colorimetric kits (Jian Cheng Bioengineering, Nanjing, China).

#### 4.6.3 Histochemistry analysis

The histochemistry analysis of haematoxylin-eosin (H&E), Masson’s trichrome and immunohistochemistry stainings was performed in this study according to a previous methodology[31]. After sacrificing the mice, the eWAT was removed and fixed with 4% paraformaldehyde at 4°C overnight, and then eWAT was embedded in paraffin, sectioned, and stained with the corresponding dyes (Beyotime, Shanghai, China) and the F4/80 antibody (ab6640; Abcam; 1:200).

#### 4.6.4 Real-time quantitative PCR (qRT-PCR)

As previously described, the total RNA of eWAT was extracted using TRIzol reagent (Thermo Fisher Scientific, Waltham, MA, USA). The cDNA was reverse transcribed, and a qRT-PCR amplification was performed using the Vazyme PCR kit (Nanjing, China) according to the manufacturer’s instructions[32]. The sequences of primers are listed in Table 3. In this study, β-actin was used as internal control, and the relative mRNA expression levels of the target genes were normalized to the β-actin levels. The results were analysed by the 2-ΔΔCt method of relative quantification.

**Table 3.** Sequences of qRT-PCR primers.

Gene	Forward Sequence	Reverse Sequence	Species
Csf1r	CCGCCTGCCTGTAAAGTGGATG	CCAGAGGAGGATGCCGTAGGAC	Mouse
Emr1	TTCCTGCTGTGTCGTGCTGTTC	GCCGTCTGGTTGTCAGTCTTGTC	Mouse
Itgb2	AGGTCGGCAAGCAACTGATTTCC	CACCAGCAGCCTCGTGACATTG	Mouse
Mki67	GCCTGCCCGACCCTACAAAATG	CTCATCTGCTGCTGCTTCTCCTTC	Mouse
Rac2	ATACCGCAGGTCAGGAGGACTATG	AACCACTTGGCACGGACATTCTC	Mouse
Tyrobp	TGACACTTTCCCAAGATGCGACTG	ATCAGCAGAGTCAACACCAAGTCAC	Mouse

### 4.7. Statistical analysis

The data were presented as the mean ± standard deviation (SD) and analysed using SPSS 23.0 and GraphPad Prism 8.0 software. The significant differences between groups were calculated by unpaired two-tailed Student’s t-test or one-way analysis of variance. A *p*-value < 0.05 was considered to be statistically different.

## 5. Conclusions

In summary, in this study, six hub genes, including Mki67, Rac2, Itgb2, Emr1, Tyrobp and Csf1r were identified which could be used as therapeutic biomarkers for obesity. In addition, the up-regulated ODEGs identified in this study were related to inflammatory response, chemokine signalling pathway and ECM–receptor interaction. Overall, these results suggest that inflammation and fibrosis could be important pathological alterations associated with the development of obese eWAT.

## Supplementary Material

Supplemental Material

## Data Availability

All of the data are presented in the manuscript.
