# Supplementary material for: Identification of major hub genes involved in high-fat diet-induced obese visceral adipose tissue based on bioinformatics approach
Source: Adipocyte. 2023 Feb 1;12(1):2169227. doi: 10.1080/21623945.2023.2169227 (PMC9897782; doi:10.1080/21623945.2023.2169227)
Supplement: Supplemental Material [file KADI_A_2169227_SM7536.zip › Supplementary Table S1.doc]

**Supplementary Table S1.** Basic information of GSE data.

| **GSE** **number** | **Background**  **strain** | **HFD**  **composition** | **Age of**  **HFD feeding** | **Weeks of**  **HFD** |
| --- | --- | --- | --- | --- |
| GSE30247 | C57BL/6J | 60% of calories from fat | 4-week old | 8 weeks |
| GSE167311 | C57BL/6J | 60% of calories from fat | Not provided | 8 weeks |
| GSE79434 | C57BL/6J | 60% of calories from fat | 7-week old | 8 weeks |
